# CD1d- and PJA2-related immune microenvironment differs between invasive breast carcinomas with and without a micropapillary feature

**DOI:** 10.1186/s12885-018-5221-9

**Published:** 2019-01-16

**Authors:** Naoki Kanomata, Junichi Kurebayashi, Yoshikazu Koike, Rin Yamaguchi, Takuya Moriya

**Affiliations:** 1 Department of Pathology, Kawasaki Medical School, Matsushima 577, Kurashiki, Okayama, 701-0192 Japan; 2 Department of Breast and Thyroid Surgery, Kawasaki Medical School, Kurashiki, Okayama, Japan; 3 Department of Pathology and Laboratory Medicine, Kurume University Medical Center, Kurume, Fukuoka, Japan; 4 Department of Pathology, Kurume University School of Medicine, Kurume, Fukuoka, Japan

**Keywords:** Invasive micropapillary carcinoma, DNA microarray, CD1D, PJA2, Immunohistochemistry, Immune microenvironment

## Abstract

**Background:**

Invasive micropapillary carcinoma (IMPC) of the breast is characterized by its unique morphology and frequent nodal metastasis. However, the mechanism for development of this unique subtype has not been clearly elucidated. The aim of this study was to obtain a better understanding of IMPC.

**Methods:**

Using representative cases of mixed IMPC, mRNA expression in the micropapillary area and usual invasive area was compared. Then, immunohistochemical analyses for 294 cases (76 invasive carcinomas with a micropapillary feature [ICMF] and 218 invasive carcinomas without a micropapillary feature [ICNMF]) were conducted. Clinicopathological analyses were also studied.

**Results:**

DNA microarray analyses for mixed IMPC showed that BC-1514 (C21orf118) was commonly upregulated in the micropapillary area. CAMK2N1, CD1d, PJA2, RPL5, SAMD13, TCF4, and TXNIP were commonly downregulated in the micropapillary area. Immunohistochemically, we confirmed that BC-1514 was more upregulated in ICMF than in ICNMF. CD1d and PJA2 were more downregulated in ICMF than ICNMF. All patients with cases of PJA2 overexpression survived without cancer recurrence during the follow-up period, although the differences for disease-free (*p* = 0.153) or overall survival (*p* = 0.272) were not significant.

**Conclusions:**

The CD1d- and PJA2-related tumour microenvironment might be crucial for IMPC. Further study of the immune microenvironment and micropapillary features is warranted.

## Background

Invasive micropapillary carcinoma (IMPC) of the breast is characterized by its unique morphology; formation of micropapillae within clear spaces separated by fibrous stroma, and reverse polarity [1]. A reverse polarity is shown by immunohistochemistry of EMA [2], MUC1 [3], sialyl Lewis X [4], and p120 catenin [5]. IMPC is known to have higher lymph vessel tumour embolus and nodal metastasis than invasive carcinoma of no special type (ICNST) [2]. Tumour-infiltrating lymphocytes [6], p63 [7], involucre [7], 34βE12 [7], stromal cell-derived factor-1 [8], CXCR4 [8], caveolin-1 [9], CD44 [10], prostate stem cell antigen [11], and LZTS1 [12] have been reported for IMPC. Marchiò et al. studied the comparative genomic hybridization analysis of pure IMPC [13] and concluded that high-level gain/amplification of 8p12–p11, 8q12, 8q13, 8q21, 8q23, 8q24, 17q21, 17q23, and 20q13 were significantly associated with IMPCs. However, to our knowledge, DNA microarray analysis of IMPC has not yet been reported. The aim of this study was to obtain better understanding of IMPC using DNA microarray analysis followed by immunohistochemistry.

## Methods

### DNA microarray

**Fig. 1:**
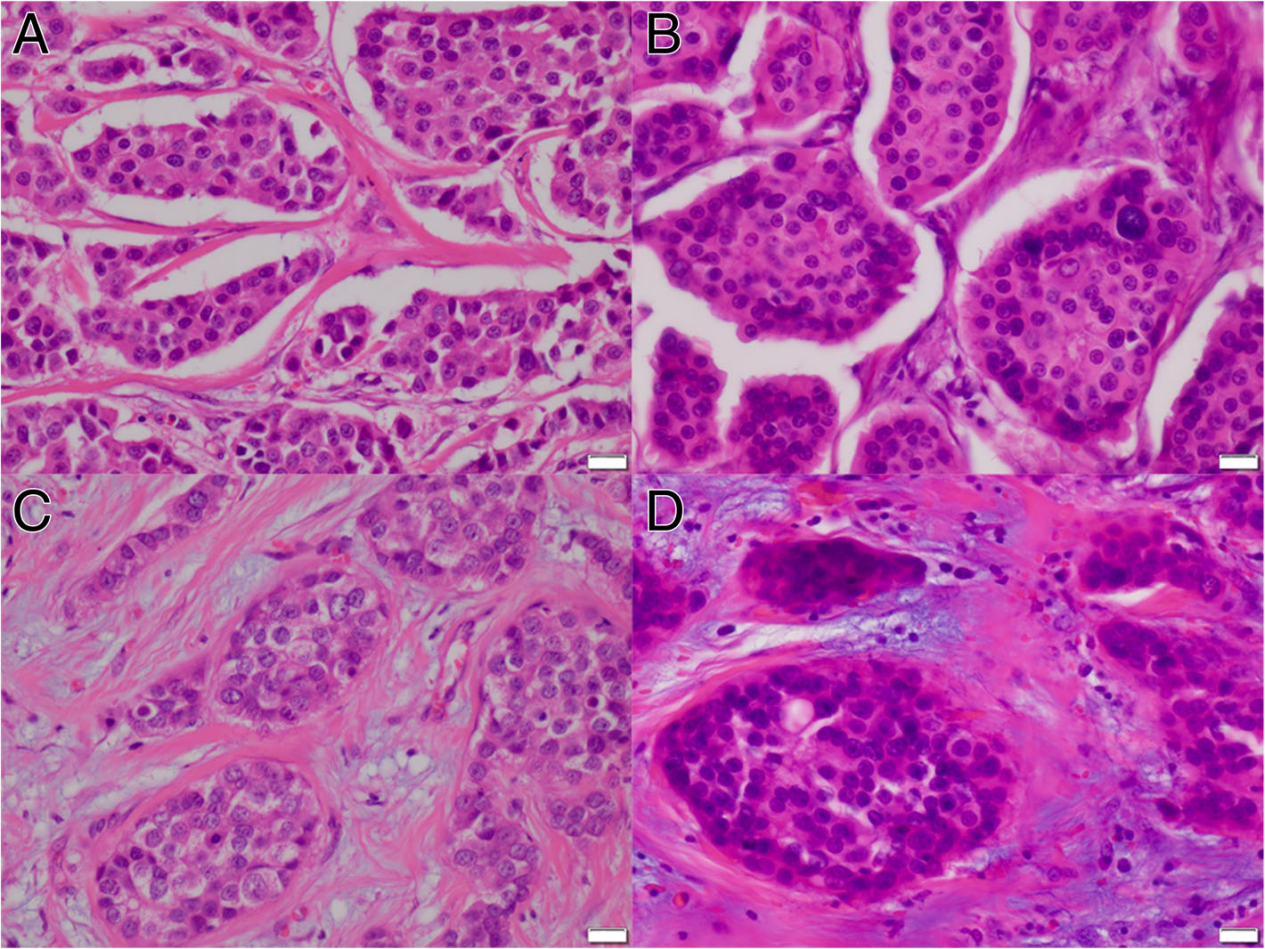
Invasive micropapillary component of mixed IMPC, case 1 (**a**). Invasive micropapillary component of mixed IMPC, case 2 (**b**). Usual invasive carcinoma component of mixed IMPC, case 1 (**c**). Usual invasive carcinoma component of mixed IMPC, case 2 (**d**). Scale bars represent 20 μm

After approval by the Institutional Review Board of Kawasaki Medical School ethics committee (approval number 909 and 2136), two representative cases of mixed IMPC were extracted from the database of the Department of Pathology, Kawasaki Medical School. Case 1 was ER–, PgR–, and HER2 3+, with histologic grade 2 and pT2N3aM0 (Fig. 1a and c). Case 2 was ER+, PgR–, and HER2–, with histologic grade 2 and pT1cN0M0 (Fig. 1b and d). The micropapillary structure was confirmed with EMA and MUC1. Paraffin sections were cut from the blocks and deparaffinized. They were stained with toluidine blue. Under microscopy, the tissue was separated to the IMPC area and the ICNST area of each case by scalpal blade. RNA was extracted and hybridization on a 3D-Gene Human Oligochip (Toray, Tokyo, Japan). The microarray data from the IMPC and ICNST areas were compared.

### Antibodies

Polyclonal antibodies were generated for SAMD13, TCF4 and TXNIP using synthetic peptides of their specific amino acid structures: positions 31–46 for SAMD13; positions 756–773 for TCF; and positions 333–351 for TXNIP. Commercially available rabbit polyclonal antibodies were used for BC-1514 (C15; Santa Cruz Biotechnology, Dallas, TX, USA), BC-1514 (W12; Santa Crus Biotechnology), CAMK2N1 (GenTex, Irvine, CA, USA), PJA2 (Abcam, Cambridge, UK), and RPL5 (Abcam). A commercial mouse monoclonal antibody was used for CD1D (clone NOR3.2 [NOR3.2/13.17], Abcam).

### Tissue microarray

The first tissue microarray was constructed with 231 consecutive surgical cases of invasive breast cancers from Kawasaki Medical School Hospital from September 2009 to December 2010. The first microarray contained 13 cases of invasive carcinoma with a micropapillary feature (ICMF: pure or mixed IMPC and ICNST with a focal micropapillary feature). The second tissue microarray was constructed with 63 cases of ICMF from January 2011 to December 2014. A KIN-2 system (Azumaya, Tokyo, Japan) with a 2-mm needle was used for the tissue microarray. For cases of ICMF, only the micropapillary area was sampled. ER and PgR were judged using 1% cutoff [14]. For HER2, HercepTest 3+ or HercepTest 2+ and FISH positive were regarded as positive [15].

### Immunohistochemistry

Immunostaining was performed using an EnVision Plus kit (Dako, Glostrup, Denmark). We cut 4 μm sections from the microarray tissue. After dewaxing and hydration, they were placed in a bath of hot citrate buffer, pH 6.0 at 95 °C for 40 min for BC-1514 (C15), BC-1514 (W12), CAMK2N1, CD1D, PJA2, SAMD13, TCF4 and TXNIP, or of Target Retrieval Solution, pH 9.0 (Dako) for RPL5 at 95 °C for 40 min. The sections were incubated with the primary antibodies for overnight at 4 °C. The dilutions of primary antibodies were: 1:200 for BC-1514 (C15), 1:100 for BC-1514 (W12), 1:500 for CAMK2N1, 1:300 for CD1D, 1:100 for PJA2, and 1:1000 for RPL5. For SAMD13, TCF4, and TXNIP, the antibodies were used at 2 μg/ml. The chromogen used was 3,3′ -diaminobenzidine tetrachloride, and the sections were counterstained with hematoxylin.

Immunohistochemistry was analysed using a histoscore that was calculated by multiplying the positive area (%) and intensity (0–3: 0 for negative, 1 for weak, 2 for moderate, and 3 for strong staining). Immunohistochemical analyses were evaluated in blinded manner.

### Statistical analyses

Statistical analyses were performed using IBM SPSS Statistics for Windows (version 25; IBM Corp., Armonk, NY). *p* <  0.05 was considered significant.

## Results

### DNA microarray

The commonly up- and downregulated factors in IMPC compared with ICNST by DNA microarray are shown in Table 1 and Table 2. BC-1514 (C21orf118) is the only gene that showed over threefold increasing expression in the IMPC area compared with the ICNST area. SAMD13, CAMK2N1, TCF4, TXNIP, RPL5, PJA2, and CD1d showed over threefold decreasing expression in the IMPC area compared with the ICNST area.

**Table 1:**
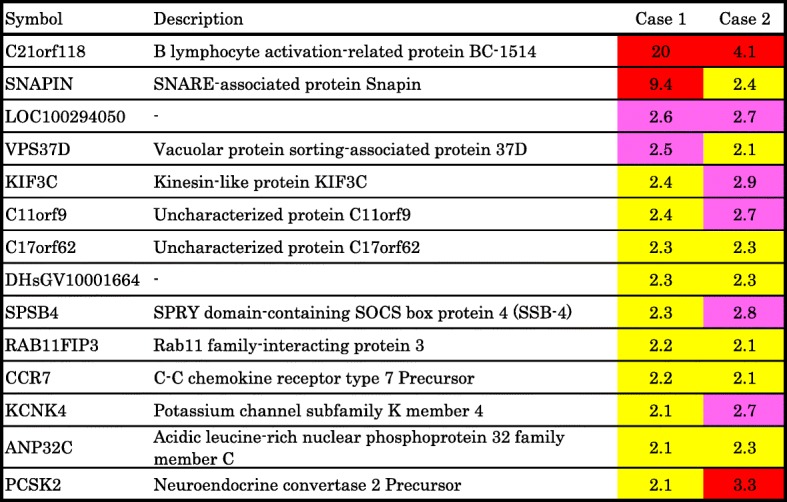
DNA microarray data of mixed IMPC. Factors more commonly upregulated in the IMPC than ICNST areas. The ratio of the expression in IMPC area to the expression in ICNST area was shown as heat map. Red column represents over 3.0, pink represents 2.5 to 3.0, yellow represents 2.1 to 2.4

**Table 2:**
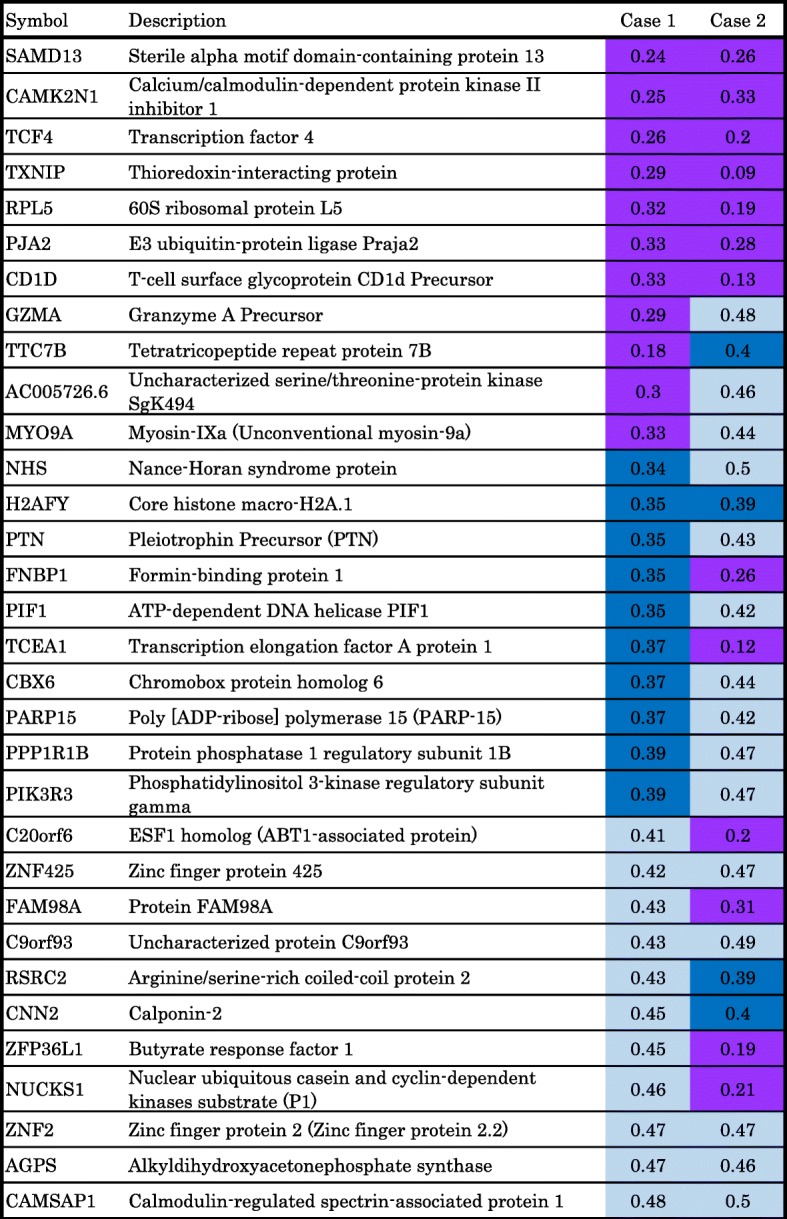
DNA microarray data of mixed IMPC. Factors more commonly downregulated in the IMPC than ICNST areas. The ratio of the expression in IMPC area to the expression in ICNST area was shown as heat map. Violet column represents under 0.34, blue represents 0.34 to 0.4, light blue represents 0.41 to 0.5

### Immunohistochemistry

**Fig. 2:**
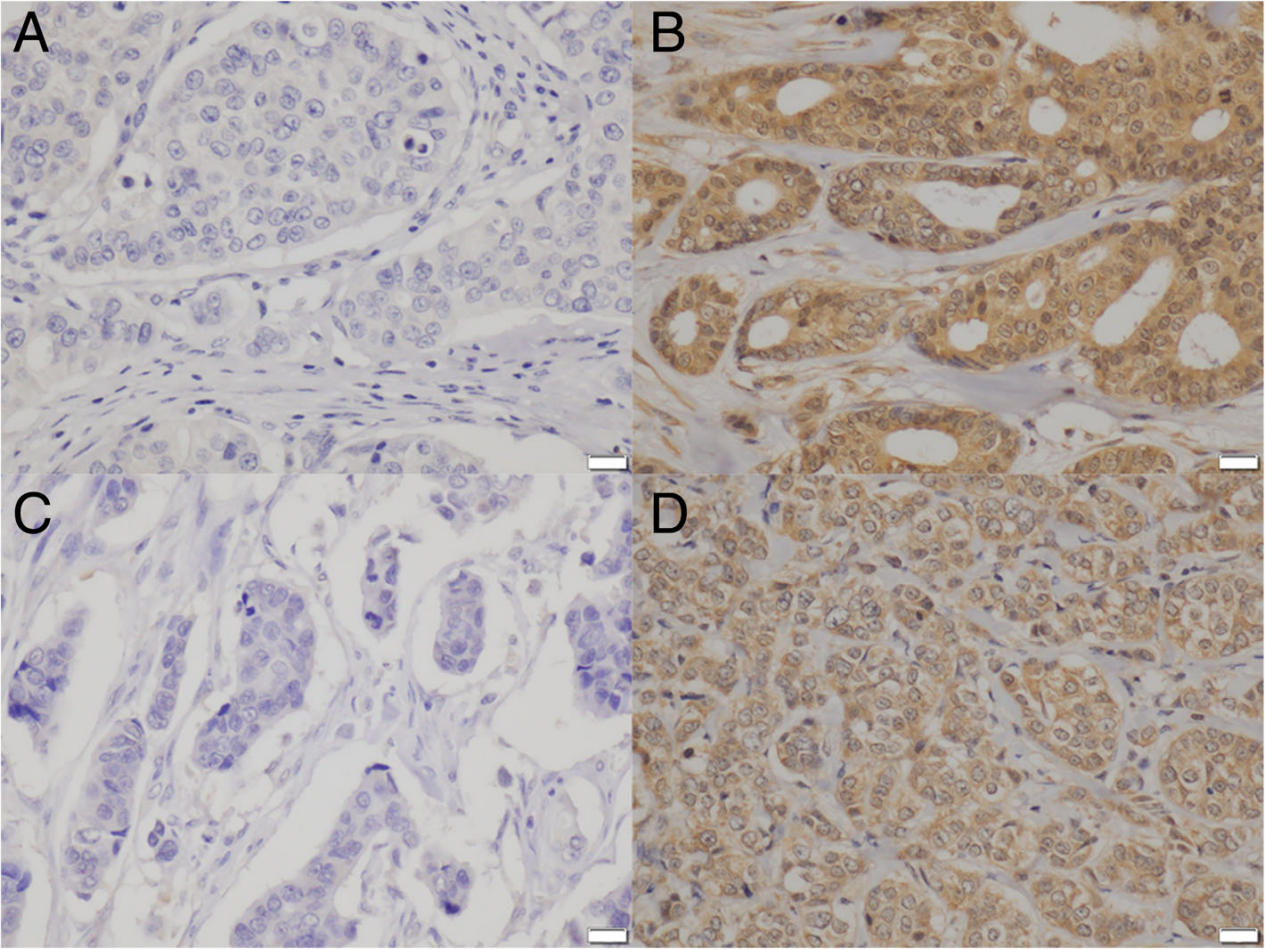
Immunohistochemistry. Negative staining for CD1d in IMPC (**a**). Diffuse staining for CD1d in ICNST (**b**). Negative staining for PJA2 in IMPC (**c**). Diffuse staining for PJA2 in ICNST (**d**). Scale bars represent 20 μm

The immunohistochemical expression of BC-1514 (C15), BC-1514 (W12), CAMK2N1, CD1d, PJA2, RPL5, SAMD13, TCF4, and TXNIP are summarized in Table 3. BC-1514 (C15), BC-1514 (W12), CD1d, and PJA2 showed results concordant with the DNA microarray (Fig. 2A–D). CAMK2N1, RPL5, SAMD13, and TCF4 showed contradictory results, and TXNIP did not show a significant difference. We have not investigated these five genes further.

**Table 3:** Immunohistochemical comparison of ICMF and ICNMF. Median (mean, IQR), * *p* <  0.05

	BC-1514 (C15)	BC-1514 (W12)	CAMK2N1	CD1d	PJA2
ICMF	200 (218.5, 200–300)	200 (243.6, 200–300)	200 (192.1, 200–200)	80 (90.6, 35–170)	100 (134.5, 90–200)
ICNMF	180 (139.1, 90–200)	200 (210.9, 180–300)	90 (83.0, 80–100)	95 (114.1, 80–190)	200 (164.3, 100–200)
*p*	< 0.001*	0.001*	< 0.001*	0.006*	0.002*
	RPL5	SAMD13	TCF4	TXNIP	
ICMF	300 (233.4, 300–300)	90 (95.8, 60–100)	200 (241.5, 200–300)	100 (126.9, 80–200)	
ICNMF	40 (58.9, 10–90)	80 (67.3, 10–90)	200 (219.2, 200–300)	90 (114.4, 80–190)	
*p*	< 0.001*	< 0.001*	0.014*	0.109

The expression of CD1d by ICNMF is similar to that of normal breast tissue (*p* = 0.373). However, expression of CD1d by ICMF is lower than that of normal breast tissue (*p* = 0.008). The expression of PJA2 by ICNMF is higher than that of normal breast tissue (*p* <  0.001). The expression of PJA2 by ICMF is similar to that of normal breast tissue (*p* = 0.259).

The clinicopathological analyses of BC-1514 (C15), BC-1514 (W12), CD1d, and PJA2 are shown in Table 4. Pathological T factor showed a significant positive correlation with BC-1514 (W12) (*p* = 0.015). Histological tumour grade and HER2 showed positive correlations with BC-1514 (W12), respectively (*p* = 0.009 and *p* = 0.043). The cases with a high Ki-67 index showed higher BC-1514 (C15) (*p* = 0.010), BC-1514 (W12) (*p* = 0.011), and CD1d (*p* = 0.036) than the cases with a low Ki-67 index.

**Table 4:** The clinicopathological analyses of BC-1514 (C15), BC-1514 (W12), CD1d, and PJA2. Median (average, IQR), * *p* <  0.05

Parameter		Cases (percentage)	BC-1514 (C15)	*P* value	BC-1514 (W12)	P value	CD1d	P value	PJA2	P value
Age										
	< 55	115 (39.1)	180 (160.7, 90–200)	0.903	200 (212.4, 200–300)	0.245	90 (98.2, 60–180)	0.182	180 (157.3, 90–200)	0.956
	≥ 55	179 (60.9)	180 (162.5, 100–200)		200 (225.7, 200–300)		95 (113.8, 60–180)		180 (155.5, 90–200)	
pT										
	I-II	281 (95.6)	180 (161.1, 90–200)	0.368	200 (218.2, 200–300)	0.015*	90 (108.1, 60–180)	0.413	180 (155.9, 90–200)	0.445
	III-IV	13 (4.4)	200 (177.5, 100–300)		300 (266.7, 200–300)		90 (100.0, 10–180)		200 (163.8, 100–200)	
pN										
	pN0	192 (65.3)	180 (163.9, 90–200)	0.709	200 (221.6, 200–300)	0.979	90 (109.8, 70–190)	0.466	180 (157.3, 90–200)	0.99
	pN1 or above	102 (34.7)	180 (157.9, 95–200)		200 (218.9, 200–300)		90 (103.9, 40–180)		180 (154.1, 90–200)	
Distant metastasis										
	M0	289 (98.3)	180 (160.5, 90–200)	0.121	200 (219.4, 200–300)	0.09	90 (108.1, 60–180)	0.696	180 (155.8, 90–200)	0.222
	M1	5 (1.7)	250 (215.0, 105–300)		300 (266.7, 200–300)		90 (92.0, 45–135)		200 (180.0, 150–200)	
pStage										
	I-II	247 (84.0)	180 (161.5, 90–200)	0.525	200 (220.1, 200–300)	0.448	90 (107.2, 60–180)	0.868	180 (155.3, 90–200)	0.416
	III-IV	47 (16.0)	200 (162.9, 100–200)		200 (222.5, 200–300)		90 (110.7, 50–190)		200 (161.0, 100–200)	
Histological grade										
	1 or 2	230 (78.2)	180 (158.7, 90–200)	0.176	200 (214.1, 200–300)	0.009*	90 (107.4, 60–180)	0.832	180 (158.2, 95–200)	0.498
	3	64 (21.8)	200 (172.6, 100–200)		200 (242.4, 200–300)		95 (109.2, 60–180)		180 (149.1, 90–200)	
Lymphatic vessel invasion										
	absent	128 (43.5)	180 (156.1, 90–200)	0.105	200 (218.8, 200–300)	0.782	90 (110.8, 70–190)	0.524	180 (156.4, 90–200)	0.69
	present	166 (56.5)	200 (166.3, 100–200)		200 (221.8, 200–300)		90 (105.4, 50–180)		180 (156.1, 100–200)	
Blood vessel invasion										
	absent	243 (82.7)	180 (163.4, 100–200)	0.57	200 (220.4, 200–300)	0.895	90 (108.4, 60–180)	0.836	180 (157.5, 90–200)	0.543
	present	51 (17.3)	180 (154.3, 90–200)		200 (220.9, 200–300)		90 (104.7, 55–145)		180 (150.0, 100–200)	
ER										
	negative	48 (16.3)	200 (157.4, 90–200)	0.97	200 (241.7, 200–300)	0.066	95 (121.7, 90–180)	0.127	200 (165.9, 95–200)	0.226
	positive	246 (83.7)	180 (162.5, 100–200)		200 (217.1, 200–300)		90 (104.9, 60–180)		180 (154.3, 90–200)	
PgR										
	negative	96 (32.7)	180 (167.2, 90–200)	0.605	200 (229.5, 200–300)	0.208	90 (110.9, 60–180)	0.632	200 (164.4, 97.5–200)	0.105
	positive	198 (67.3)	180 (159.5, 100–200)		200 (216.7, 200–300)		90 (106.3, 60–190)		180 (152.4, 90–200)	
HER2										
	negative	247 (85.2)	180 (160.1, 90–200)	0.161	200 (216.8, 200–300)	0.043*	90 (108.2, 65–190)	0.471	180 (154.4, 90–200)	0.207
	positive	43 (14.8)	200 (176.0, 100–200)		300 (247.6, 200–300)		92.5 (108.3, 40–180)		180 (162.4, 95–200)	
Ki-67										
	< 14	68 (23.1)	160 (136.8, 90–200)	0.010*	200 (196.7, 180–250)	0.011*	90 (91.7, 55–92.5)	0.036*	180 (150.8, 90–200)	0.603
	≥ 14	205 (69.7)	180 (158.9, 95–200)		200 (225.1, 200–300)		95 (112.8, 60–190)		180 (159.5, 90–200)	
Triple negative										
	no	263 (89.5)	180 (164.4, 100–200)	0.095	200 (219.4, 200–300)	0.434	90 (106.3, 60–180)	0.106	180 (154.9, 90–200)	0.199
	yes	31 (10.5)	120 (135.8, 70–200)		200 (231.2, 200–300)		97.5 (121.5, 90–190)		200 (167.8, 90–200)	

### Survival analyses

**Fig. 3:**
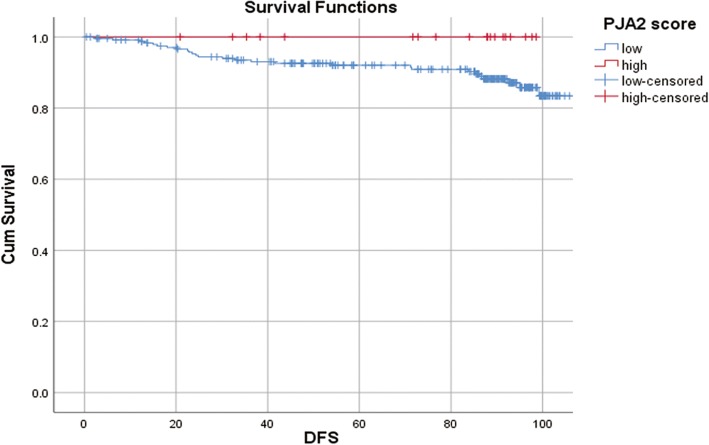
Kaplan–Meier analysis of disease-free survival. No patients with cases having overexpression of PJA2 (> third quadrant) died or had tumour recurrence during the observed period (*p* = 0.153)

The median follow-up periods for disease-free survival was 87.8 months (range, 0.4 to 113.7 months) and for overall survival was 88.2 months (range, 0.4 to 119.27 months). The univariate Cox hazard survival analyses are shown in Table 5. pT, pN, and pathological stage were significant for disease free survival (*p* = 0.001, < 0.001 and 0.011). pT, pN, stage, histological grade, and PgR were significant for overall survival (*p* <  0.001, 0.004, < 0.001, 0.001, and 0.005, respectively). BC-1514 (C15, W12), CD1d, and PJA2 did not show any significant difference for disease-free survival or overall survival. All patients with cases having high PJA2 survived without cancer recurrence (19 of 19) using the third quadrant as a cut-off value, but there was no significant difference (*p* = 0.153 for disease-free survival [Fig. 3] and *p* = 0.272 for overall survival).

**Table 5:** Univariate Cox proportional hazards model

		Disease free survival		Overall survival	
Parameter	Criteria	*p*	HR (95% CI)	*p*	HR (95% CI)
Age	< 55 vs. 55 or more	0.556	0.803 (0.386–1.669)	0.322	0.594 (0.212–1.667)
pT	I-II vs. III-IV	0.001*	6.135 (2.123–17.86)	< 0.001*	9.090 (3.247–25.64)
pN	0 vs. 1–3	< 0.001*	4.950 (2.252–10.87)	0.004*	4.150 (1.560–11.11)
pStage	I-II vs. III-IV	0.011*	2.793 (1.267–6.135)	< 0.001*	5.587 (2.212–14.08)
Histological grade	1/2 vs. 3	0.360	1.464 (0.648–3.307)	0.001*	4.782 (1.887–12.12)
ER	Neg. vs. pos.	0.934	0.960 (0.366–2.519)	0.528	0.698 (0.230–2.123)
PgR	Neg. vs. pos.	0.142	0.770 (0.578–1.202)	0.005*	0.246 (0.092–0.656)
Ki-67	< 14% vs. 14% or more	0.237	1.912 (0.653–5.602)	0.310	2.171 (0.486–9.702)
HER2	Neg. vs. pos.	0.600	1.377 (0.416–4.553)	0.779	1.194 (0.335–4.132)
Intrinsic subtype	Triple negative vs. others	0.236	1.792 (0.683–4.695)	0.115	2.445 (0.805–7.463)
IMP	With IMP vs. without IMP	0.064	2.032 (0.958–4.309)	0.308	1.669 (0.624–4.462)
BC-1514 (C15)	< 180 (median) vs. 180 or more	0.476	1.342 (0.597–3.016)	0.304	1.823 (0.580–5.726)
BC-1514 (W12)	< 200 (median) vs. 200 or more	0.613	1.290 (0.480–3.468)	0.365	1.985 (0.451–8.739)
CD1d	< 90 (median) vs. 90 or more	0.674	0.843 (0.381–1.866)	0.391	0.609 (0.196–1.890)
PJA2	< 180 (median) vs. 180 or more	0.454	0.753 (0.357–1.584)	0.742	0.847 (0.315–2.276)

*p < 0.05

## Discussion

CD1d-presents lipid antigens to activate natural killer T (NKT) cells, through the interaction with the T-cell receptor present on NKT membranes [16]. NKT cells display antitumour immune responses when activated by the synthetic glycosphingolipid, α-galactosylceramide (αGalCer) [16]. CD1d expression was reported in the intestines, liver, pancreas, kidney, uterus, skin, conjunctiva, thymus, tonsil, and breast [17]. We demonstrated that CD1d expression is lower in ICMF than in ICNMF and normal breast tissue. Downregulation of CD1d might be important for IMPC to avoid an NKT cell antitumour response. Hix et al. showed that downregulation of CD1d inhibited NKT-mediated antitumour immunity and promoted metastasis of breast cancer in vitro and in vivo [18]. Ascierto et al. reported that CD1d and CD96 were good prognostic factors for breast cancer [19], although we could not show a significant correlation between prognosis and CD1d expression. To improve the immune microenvironment, strategies such as αGalCer [20] administration may be a therapeutic option for breast cancer.

PJA2, also known as PRAJA2, regulates the protein kinase A signal strength and duration in response to cAMP [21]. PJA2 increases the accumulation of ubiquitylated malignant fibrous histiocytoma amplified sequence 1, which promotes M1 macrophage polarization and M2 to M1 macrophage transformation [22]. M1 macrophage promotes antitumour immunity, while M2 macrophage promotes tumour progression [23]. We showed lower expression of PJA2 in ICMF than ICNMF by both DNA microarray and immunohistochemistry. In IMPC, M2 macrophage polarization by a shortage of PJA2 might facilitate tumour progression.

CD1d, PJA2, and granzyme A precursor also showed lower expression in IMPC than in ICNST in our DNA microarray screening. Granzyme A, an enzyme present in cytotoxic T lymphocytes, has tumouricidal activity [24, 25]. The potential of IMPC to evade the immune system may be supported by multiple mechanisms.

We demonstrated BC-1514 mRNA upregulation in IMPC and greater immunohistochemical expression in ICMF than ICNMF. However, the record of BC-1514 for *Homo sapiens* has been withdrawn by the National Center for Biotechnology Information because of insufficient evidence (https://www.ncbi.nlm.nih.gov/gene/378829, last accessed on June 6, 2018). Our immunohistochemistry for BC-1514 probably showed some cross reaction to an unknown substance.

One of the limitations of this study is that our DNA microarray data was obtained from only two mixed IMPC cases. We thought that the candidate markers for micropapillary feature were well narrowed down by just two cases. However, four (CAMK2N1, RPL5, SAMD13, and TCF4) of eight markers showed contradictory results, and TXNIP did not show a significant difference by immunohistochemistry. Additional DNA microarray would be promising to increase the accuracy, and should have improved the efficiency of our immunohistochemical study. Our DNA microarray data should be interpreted with caution.

## Conclusions

Our present study suggests the CD1d- and PJA2-related tumour microenvironment might be crucial for IMPC. Further study of the immune microenvironment and micropapillary feature is warranted.

## Abbreviations

ICMF: Invasive carcinoma with a micropapillary feature
ICNMF: Invasive carcinoma without a micropapillary feature
ICNST: Invasive carcinoma of no special type
IMPC: Invasive micropapillary carcinoma
IQR: Interquartile range
